# Spontaneous Generation

**Published:** 1842-06-18

**Authors:** 


*Spontaneous Generation.*—Our readers may remember the excitement caused in the year 1837, by the announcement that Mr. Crosse, of Broomfield, had observed the development of certain insects incident to the long-continued action of voltaic pairs. Little additional information on this mysterious subject has since transpired until Tuesday, the 15th instant, when a paper from Mr. Weekes, of Sandwich, was read before the London Electrical Society, detailing a successful repetition of Mr. Crosse's experiments. Among the cavilling which arose in connexion with the original experiments the possibility was urged that the ovæ of the insects might be in the air. Mr. Weekes' experiments were so conducted, that this objection can be scarcely tenable. A well-charred block of beech, containing a circular groove to receive a bell-glass, was the base of the instrument. The groove was filled with mercury. A tumbler, containing the solution of silicate of potass, was beneath the bell. The silica was obtained by subjecting to a furnace heat a piece of fine black flint obtained out of the centre of a "bowlder," selected from amongst those lying on the shore at Sandwich. The silica was united to the potass by a furnace heat; the result quenched in boiling water. The solution was immediately covered, and filtered under cover. All things being prepared, the voltaic current was sent through the solution on the 3d of December, 1841; and from that date to the present time the apparatus has not been disturbed. At the end of October, 1841, the first insect was observed. On the 16th of November five were discovered. Since that date insects have been repeatedly seen. We must not omit to mention that the bell-glass was placed in total darkness, the screen being only removed when the progress was being examined. Mr. Weekes mentioned that he has another apparatus in action, very similar to this, with the exception that the bell was filled with oxygen, and expressed an anticipation that he should sooner or later detect insect life there. This expectation was realized a few days ago. In an appendix to his communication, bearing date February 27, 1842, he states that on the previous morning he "perceived eight or ten full grown acari in vigorous locomotion on the inner surface of the air-bell."—*London Times.*

